# Transcranial alternating current stimulation (tACS): from basic mechanisms towards first applications in psychiatry

**DOI:** 10.1007/s00406-020-01209-9

**Published:** 2020-11-19

**Authors:** Osama Elyamany, Gregor Leicht, Christoph S. Herrmann, Christoph Mulert

**Affiliations:** 1 Centre of Psychiatry, Justus-Liebig University, Klinikstrasse 36, 35392 Giessen, Hessen Germany; 2 Centre for Mind, Brain and Behaviour (CMBB), University of Marburg and Justus-Liebig University Giessen, Marburg, Germany; 3 Department of Psychiatry and Psychotherapy, University Medical Centre Hamburg-Eppendorf, Hamburg, Germany; 4 Experimental Psychology Lab, Centre for Excellence “Hearing4all,” European Medical School, University of Oldenburg, Oldenburg, Lower Saxony Germany; 5 Research Centre Neurosensory Science, University of Oldenburg, Oldenburg, Lower Saxony Germany

**Keywords:** Transcranial alternating current stimulation (tACS), Non-invasive brain stimulation (NIBS), Psychiatry, Schizophrenia, Depression, OCD

## Abstract

Transcranial alternating current stimulation (tACS) is a unique form of non-invasive brain stimulation. Sinusoidal alternating electric currents are delivered to the scalp to affect mostly cortical neurons. tACS is supposed to modulate brain function and, in turn, cognitive processes by entraining brain oscillations and inducing long-term synaptic plasticity. Therefore, tACS has been investigated in cognitive neuroscience, but only recently, it has been also introduced in psychiatric clinical trials. This review describes current concepts and first findings of applying tACS as a potential therapeutic tool in the field of psychiatry. The current understanding of its mechanisms of action is explained, bridging cellular neuronal activity and the brain network mechanism. Revisiting the relevance of altered brain oscillations found in six major psychiatric disorders, putative targets for the management of mental disorders using tACS are discussed. A systematic literature search on PubMed was conducted to report findings of the clinical studies applying tACS in patients with psychiatric conditions. In conclusion, the initial results may support the feasibility of tACS in clinical psychiatric populations without serious adverse events. Moreover, these results showed the ability of tACS to reset disturbed brain oscillations, and thus to improve behavioural outcomes. In addition to its potential therapeutic role, the reactivity of the brain circuits to tACS could serve as a possible tool to determine the diagnosis, classification or prognosis of psychiatric disorders. Future double-blind randomised controlled trials are necessary to answer currently unresolved questions. They may aim to detect response predictors and control for various confounding factors.

## Introduction

Transcranial alternating current stimulation (tACS) is a widely used non-invasive brain stimulation (NIBS) method. It has been used for more than a decade in different fields, such as cognitive neuroscience [1, 2]. However, its use in psychiatric clinical research began with case reports [3], and only recently have the first well-structured double-blind randomized controlled trials examined its efficacy for the treatment of psychiatric disorders [4]. tACS involves direct delivery of alternating electric currents to the scalp. The current travels through the skull to affect mostly cortical neurons. Such alternating current has a sinusoidal waveform where the voltage changes gradually from positive to negative every half-cycle. Therefore, the current flows from an anodal electrode to a cathodal electrode in one half-cycle and in the reverse direction in the second half-cycle [5].

The concept underlying alternating current is to simulate the naturally occurring rhythmic pattern of electrophysiological activity of the brain, which can be detected by electroencephalography (EEG) and magnetoencephalography (MEG) [6]. Such rhythmic patterns, oscillating at a certain frequency, are called brain oscillations. Various specific brain oscillations have been coupled with diverse brain functions and states [7]. Moreover, connectivity and communication between distant cortical regions were shown to be associated with the synchronization of brain oscillations within these regions [8, 9]. In that sense, tACS is also used to couple or decouple two connected neuronal circuits by synchronizing or desynchronizing their oscillations, respectively. Accordingly, tACS might represent a potential therapeutic tool by modifying altered brain oscillations and connectivity patterns that were previously identified in various psychiatric disorders.

The typical setup of tACS involves the application of electrodes onto the scalp, whose position and size can be modified to specifically target a certain brain region [10, 11]. For this purpose, positioning of the electrodes is designed according to computational models to optimize the stimulation parameters [12, 13]. Furthermore, the parameters of the alternating current itself can be customized in terms of frequency, amplitude, phase shape, phase timing, and the duration and number of stimulation sessions. The stimulation frequency is usually set to EEG frequencies to modulate the brain processes associated with them. Other parameters may vary according to the study question and brain electric activity to be modulated (some examples are discussed below; for more details, see this review: [14]).

## Mechanism of action

Although the exact mechanisms of action of tACS are still not well understood, there is growing evidence of possible explanations. Electrophysiological methods have been extensively used to elucidate these mechanisms, as well as the effects of tACS, mostly EEG and, to a lesser degree, MEG, in humans, and intracranial and local field potential recordings in animals [15, 16]. Those methods have shown two main categories of tACS effects: online effects (those that coincide with the stimulation duration), and offline effects or after-effects (those that outlast the stimulation period). Both involve entrainment of brain oscillations to the stimulation frequency and coupling or decoupling of long-range oscillatory connectivity between distant brain regions [17, 18]. To understand these effects and their underlying electrophysiological processes, we summarize previous literature bridging the gap between cellular and whole network levels.

Upon tACS application to the skull, some of this alternating current reaches the brain. As a result, it causes the cell bodies and dendrites of the cortical neurons to alter their membrane potential towards depolarization or hyperpolarization in an oscillatory fashion [16]. This alternating change in the membrane potential is thought to be sufficient to alter the probability of a neuron generating action potentials [19]. However, it is not strong enough to change the rate of action potentials, as it controls only their timing in a frequency- and location-specific manner [20]. That is why this stimulation seems to be a type of sub-threshold stimulation that does not directly fire neuronal spikes [14, 21].

The influence of tACS depends not only on the amplitude and frequency but also on the three-dimensional orientation of both neurons and the penetrating current [22]. Its effects result from manipulating the membrane potential of neurons that are aligned with the introduced electric field, mostly pyramidal cells in layer V. These cells are extremely sensitive to current changes due to their elongated soma-dendritic axis [22, 23]. Moreover, they have characteristic properties, including intrinsic resonance, neuroplastic activity and cortico-cortical projections [24–26]. Therefore, these cells, once stimulated by tACS, show specific frequency resonance, long-term after-effects and long-range oscillatory cortical connectivity (more details on resonance and after-effects are discussed below). Similarly, the direction of the electric stimulation substantially changes the properties of the resulting field, and thus, its effects on the neurons [27].

In 2018, Liu et al. suggested five different neuronal mechanisms that could translate the previously mentioned cellular effects of tACS into whole network activity at a larger scale [28]. First, stochastic resonance: a wide range of tACS-affected neurons, differing in their momentary probability to generate action potentials, will react stochastically to be either polarized or hyperpolarized. This leads to the absence of a theoretical ‘minimum effective threshold’. Second, rhythm resonance: this occurs when the tACS frequency is the same as that of the endogenous oscillations. This ends with the stimulatory current wave striking the endogenous one at a similar phase every cycle. Third, temporal biasing of spikes: the spike timing of neurons is regulated by the interaction between the stimulation and internal currents, which may work synergistically to excite the same group of neurons during each cycle of stimulation. Fourth, network entrainment: the entrainment of an endogenous irregular activity necessitates an external current with sufficiently stronger amplitude. Finally, imposed pattern: to overcome endogenous regular oscillations and introduce a new oscillation, the strongest stimulation is required [28].

These mechanisms support the explanation by Vosskuhl et al. [14] of the large-scale effects of tACS. The authors attributed the online and offline tACS effects to two synergistic phenomena: entrainment and neuroplasticity, respectively. Entrainment, by definition, takes place when an external rhythmic system affects another naturally occurring one, forcing it to follow its own oscillating frequency. During tACS, the external driving current forces the endogenous brain oscillations to follow in terms of frequency and phase [14, 29]. Such entrainment has two crucial properties: “Arnold tongue”, and harmonics. Arnold tongues describe the relationship between the stimulation amplitude and its corresponding range of frequencies at which tACS can entrain endogenous brain oscillations. When the amplitude of this stimulation increases, it entrains brain oscillations in a wider range of frequencies [30]. Harmonics describe the preference of an intrinsic oscillator to be trained by tACS at multiple frequencies that have an n:m relationship to the endogenous frequency, e.g., at twice or half the endogenous frequency [23, 31].

For the neuroplasticity to elicit offline effects, long-term plasticity should occur in one form among two primary mechanisms: Long-term-potentiation (LTP) and long-term-depression (LTD), two results of spike-timing-dependent plasticity (STDP) [32]. LTP is the potentiation of a synaptic connection when the presynaptic action potential comes before the post-synaptic potential. In contrast, LTD is the weakening of the synapse if the presynaptic action potential follows the post-synaptic one. Therefore, these phenomena are the primary culprits that elicit offline tACS effects by increasing or decreasing neural synchronization [33]. This explanation has been confirmed by many studies [33–36], which may explain why the offline effects of tACS have been shown to last for 70 min after one stimulation session lasting 20 min [37].

From this standpoint, we try to refer to some unique features of tACS as a neuromodulator in contrast to other forms of NIBS, yet it is relatively new and the least studied form. Transcranial direct current stimulation (tDCS), a closely related type of NIBS that constantly depolarizes or hyperpolarizes the affected neurons [38], specifically changing axonal membrane potential [39]. In fact, tACS is a specific version of tDCS where the current is set to fluctuate sinusoidally between the electrodes rather than exhibiting constant polarity. Similar to tACS, tDCS effects depend on the orientation of the neurons relative to the current direction [40, 41], and it can induce both online and offline neuroplastic effects [42, 43]. Equivalently, tDCS does not directly affect the neuronal firing rate, but rather its probability and spontaneous activity via subthreshold voltage changes [44]. Although tDCS is shown to modulate oscillatory brain activity [45], tACS is more effective at entraining the endogenous brain oscillations as it mimics the alternating nature of brain oscillations [2, 14, 46] (For more details about the mechanisms of action of tDCS, see these reviews: [47–51]).

Another more studied method of NIBS is transcranial magnetic stimulation (TMS), which is approved for the treatment of some psychiatric disorders [52–54]. In TMS, a magnetic field is produced by a coil applied to the scalp and then travels through the skull to elicit electric fields in the cortical neurons [55]. Compared to tACS, TMS, a suprathreshold stimulator, produces action potentials in silent neurons [56] in the form of two successive volleys. While the first volley (direct waves) represents direct activation of pyramid tract axons, the second (indirect waves) reflects synaptic activation of the same neurons [57]. As expected, TMS evokes long-term synaptic changes and thus after-effects beyond the stimulation period [58] (For more details about the mechanisms of action of TMS, see these reviews: [59–63]). However, practically, tACS exhibits superior cost, portability, tolerability, and safety profiles [64, 65]. In other words, tACS is a feasible tool that reshapes or re-synchronizes intrinsic brain rhythms, manipulating the associated brain functions without adding extra excitatory or inhibitory burden. Given that (1) tACS clinical research is still in its infancy, and (2) tACS possesses such unique features, we aim, in this review, to encourage more tACS usage in psychiatric research.

## Altered brain oscillations and tACS applications in psychiatric disorders:

Given the association between EEG brain oscillations and various brain functions, many researchers have managed to successfully modulate normal cognitive functions by manipulating brain oscillations or connectivity patterns using tACS (for more details see these reviews [7, 14, 65–67]). Motivated by successful tACS applications in cognition, future investigations could aim to normalize pathological brain oscillations, and identify beneficial tACS roles in the management of psychiatric disorders. In this section, we focus on such electrophysiological alterations that could benefit researchers in designing tACS clinical trials in psychiatric patients. Moreover, we review previous clinical trials that have already examined the role of tACS in psychiatry. Six psychiatric disorders are discussed, where a subsection is devoted to each disorder.

It is noteworthy to state two caveats here to properly understand the current state of the literature regarding disturbed brain oscillations in psychiatry. First, this is not a comprehensive overview of disturbed brain oscillations in all psychiatric disorders, but rather, it presents six of the most studied and major disorders. Hence, this section is intended to provide only a glimpse of this interesting electrophysiological approach in psychiatric pathophysiology. Second, because of the heterogeneity of the studies and the limited knowledge of some disturbed oscillations, we tried to selectively focus on some of the more replicated and reproduced findings supported by different studies. Therefore, the following electrophysiological changes should be extrapolated with extreme caution before building around them to design tACS protocols.

**Fig. 1:**
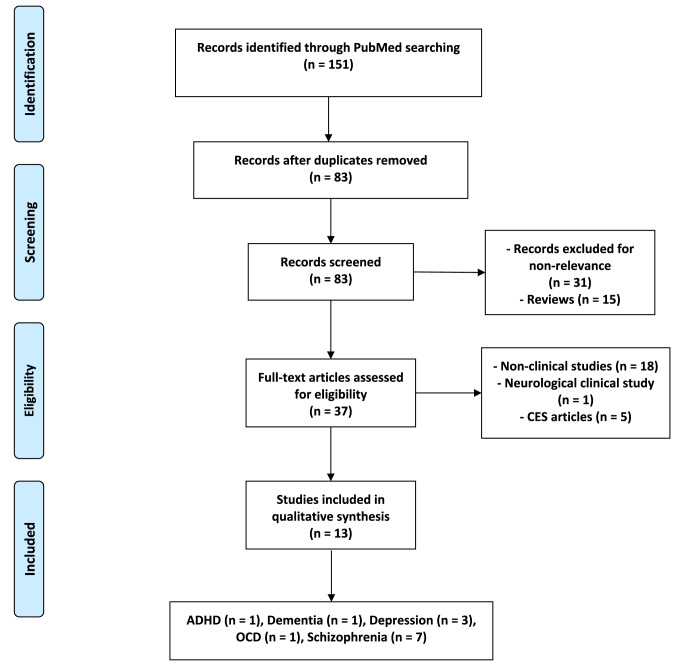
PRISMA Flow Diagram of the included articles [68]. *ADHD* attention deficit hyperactivity disorder, *CES* cranial electrotherapy stimulation, *OCD* obsessive-compulsive disorder

The results of tACS clinical studies were identified according to a systematic search on PubMed using two keywords with the Boolean operator “AND”. The first keyword was “tACS” or “alternating current stimulation” throughout the search process. The second term was changeable to signify several psychiatric disorders (“ADHD”, “Insomnia”, “Depression”, “Schizophrenia”, “Bipolar”, “OCD”, “Anxiety”, “PTSD”, “Dementia”, and “Alzheimer”). The search process, last performed in June 2020, yielded a total of 151 records, including 68 duplicates. The original 83 publications were screened to exclude 31 non-tACS relevant articles and 15 reviews. After a careful assessment of the remaining 37 studies, a stroke-related article and 18 non-clinical experiments were excluded. Five publications actually applied cranial electrotherapy stimulation (CES) rather than tACS, and thus were excluded. The remaining 13 eligible articles, which show the experimental application of tACS in patients with any psychiatric disorder, were included (see Fig. 1  [68]).

The included articles comprise study designs with different levels of evidence: three randomized double-blind controlled trials, two single-blind randomized controlled trials, an open-label non-controlled clinical trial, a longitudinal case–control study, two case series, and four case reports (see Table 1). They cover five different psychiatric disorders: one study on attention-deficit/hyperactivity disorder (ADHD), three publications on depression, seven on schizophrenia, one on dementia, and one case series on OCD. Some of the included articles conducted a relatively non-robust study design, such as case reports. However, we will consider them in further discussion due to the limited number of clinical trials and the variety of investigated psychiatric disorders. Accordingly, the reader could appreciate the shortage of evidence to apply tACS in psychiatry. After discussing the included articles for each disorder, we state some general remarks on the application of tACS in participants with psychiatric illnesses.

**Table 1:** Included articles on psychiatric applications

Publication	Study design	Participants	Stimulation protocol					Study outcomes	Main Findings
Author, Year			Sessions’ number	Session duration	Current amplitude (Peak-To-Peak)	Frequency	Electrode Location		
A. Schizophrenia									
Sreeraj et al. [125]	Case report	One patient with paranoid schizophrenia	Two sessions with 2 days apart	20 min	Session 1: 2 mA, session 2: 1 mA	Session 1: 6 Hz, session 2: 40 Hz	Left DLPFC (F3) and the left posterior parietal region (P3)	WM task	Improved performance in the WM task only with 6 Hz tACS
Sreeraj et al. [126]	Case report	One patient with paranoid schizophrenia	5 sessions for 5 consecutive days	20 min	2 mA	6 Hz	Left DLPFC (F3) and the left posterior parietal region (P3)	WM task	- Improved working memory - Improvement in other cognitive domains - Persistence of improvement for 50 days
Kallel et al. [127]	Open case series	3 clozapine-resistant patients with schizophrenia	20 daily on weekdays for 4 consecutive weeks followed by 4-week follow-up	20 min	2 mA	4.5 Hz	Both DLPFCs	SANS; PANSS; SUMD; HAMA; and side effects	Reduction in negative symptoms, anxiety and general psychopathology symptoms, and improvement in insight
Sreeraj et al. [128]	Open-label non-controlled clinical trial	12 patients with schizophrenia and persistent delusions	Twice daily for 5 days, 9 patients continued for 5 more days, and another patient continued for 4 more days	20 min	2 mA	10 Hz	Two electrodes on AFz and Cz	PSYRATS—Delusions, SAPS and SANS	- Reduction in the delusion severity, positive and negative symptoms as well as tolerance of tACS - Maintained effect for one month
Hoy et al. [129]	Single-blind randomized controlled trial	10 patients with schizophrenia	Three separate sessions at least 3 days apart	20 min	2 mA	40 Hz-tACS, tDCS and sham stimulation	Left DLFPC	WM task	No significant tACS effects
Mellin et al. [4]	Double-blind randomized controlled trial	22 patients with schizophrenia	Twice daily for 5 consecutive days	20 min	2 mA	10 Hz, tDCS or sham	Left frontal and temporal lobes	Primary outcome: AHRS, secondary outcome: PANSS, BACS	No significant results
Ahn et al. [131]	Double-blind randomized controlled trial	22 patients with schizophrenia	Twice daily for 5 consecutive days	20 min	2 mA	10 Hz, tDCS or sham	Left frontal and temporal lobes	AHRS, hdEEG	- 10 Hz-tACS showed modulated functional connectivity, and enhanced alpha oscillations and 40 Hz ASSR - This enhancement correlated with the reduction of auditory hallucinations
B. Depression									
Alexander et al. [150]	Double-blind, randomized pilot clinical trial	32 MDD patients	5 consecutive days	40 min	4 mA at Cz, 2 mA at F3 and F4	10 Hz-tACS, 40 Hz-tACS); or sham	Bifrontal: Two electrodes over F3 and F4, and a third over the vertex	Primary outcome: MADRS at 4-week follow-up, secondary: hdEEG, HDRS, BDI	- No significant primary outcome - Two weeks after completion of the intervention, the 10 Hz-tACS group had more responders compared with 40 Hz-tACS and sham groups as well as a significant reduction in alpha power over the left frontal regions at day 5
Riddle et al. [151]	Case report (extension of (Alexander et al., 2019))	One female MDD patient	12 weekly sessions	40 min	4 mA at Cz, 2 mA at F3 and F4	10 Hz-tACS	Bifrontal: Two electrodes over F3 and F4, and a third over the vertex	Daily self-reported 10-point Likert scale, and monthly MADRS	Remission (MADRS = 1 7) after twelve weeks
Wilkening et al. [152]	Case report	One female pregnant MDD patient	9 weekly sessions	20 min	2 mA	40 Hz-tACS with DC offset	Bifrontal: Two electrodes over F3 and F4	HDRS, BDI, PANAS, and TMT	Improved scores after 9 stimulations, and after two-week follow-up. Remission after three months
C. OCD:									
Klimke et al. [3]	Cases series	7 OCD treatment-resistant patients	3 session per week. Treatment duration varied across patients Follow-up duration was 58 days and one year for 4 patients and the other three, respectively	20 min	650 µA	40 Hz	Fp1-T3 and Fp2-T4	YBOCS, CGI	The symptoms improved in all patients, and the improvement lasted throughout the follow-up duration
D. ADHD									
Dallmer-Zerbe et al. [185]	Randomized single-blind controlled study	18 ADHD patients	One stimulation or Sham	20 min	1 mA	Timing and frequency personalized to coincide the stimulation peaks with P300 oscillation peaks	Multiple electrodes on the central, parietal and temporal lobes	EEG, visual oddball task	Increase in P300 amplitude in the stimulation group accompanied by a behavioural improvement defined by a decrease in omission errors
E. Dementia:									
Naro et al. [194]	Longitudinal case–control study	35 AD and 25 MCI individuals and 27 age-matched health participants	Both sham and one of five verum stimulation sessions on a weekly basis	10 min	1 mA	A range between 40 and 120 Hz at steps of 20 Hz	Left M1, PMA, SMA, DLPFC, or DMPFC	EEG and different neuropsychological tests (see text)	- Enhancement of gamma-band oscillations and a clinical improvement in MCI, but not AD patients - tACS predicted the progression from MCI to AD

### Schizophrenia

With the aid of EEG and MEG, a great bulk of research has already identified characteristic alterations of brain oscillations in patients with schizophrenia [69]. These electrophysiological fingerprints of schizophrenia have been shown to be heterogeneous but task-/state-, location, and frequency-specific, where they, in turn, correlate with the severity of certain symptoms [70]. However, such heterogeneity is not surprising given the heterogeneity of clinical presentations in schizophrenia [71]. Pertaining to the task/state aspect, three distinctive patterns of brain oscillations were identified: evoked, induced and resting-state [69]. Each type could be compromised peculiarly in schizophrenia, so tACS strategies should precisely consider which one/ones to modify.

Regarding the frequency and location domains, the results might be harmonious in the low-frequency range (alpha and theta), whereas they are not in the high-frequency range (beta and gamma). A general persistent finding in schizophrenia is reduced alpha power, especially in the resting-state, which might be linked to the increased state of arousal and abnormal self-referential processing [70, 72–76]. Delta and theta waves are mostly elevated [77–79], but only theta activity is decreased, especially in the frontal lobe [80], correlating with an impairment in working memory [81, 82]. Furthermore, theta-band connectivity was shown to be increased, especially between the frontal and parietal regions, during the resting state [83, 84]. Although beta activity in schizophrenia is not well-studied, some studies showed that it was decreased in both resting-state and task-related brain activity [85, 86]. Alterations of gamma-band oscillations have been extensively investigated as a neurobiological correlate of different stages and symptoms of schizophrenia [87] and are commonly interpreted as an imbalance between excitation and inhibition (E/I-balance), which is considered a crucial mechanism in the pathophysiology of schizophrenia [88].

A major and often replicated finding in schizophrenia is the reduction of stimulus-evoked task-related gamma power and coherence between different brain regions [85, 89–95], which, besides the chronic illness, appears across different stages and models of the disease, including in patients with their first episode of psychosis [95, 96], in healthy relatives of patients [97, 98], in subjects at high risk for developing psychosis [99], and in the ketamine model of schizophrenia [100]. Most of the studies showing reduced task-related gamma oscillations examined impaired cognitive function [101]. However, there are also reports linking increased stimulus-related gamma-synchrony to positive schizophrenia symptoms [102]. As well, in terms of gamma power, the evoked visual gamma-band response is correlated with positive and disorganised schizophrenia symptoms [103], and even with positive schizotypal personality traits [104]. Moreover, spontaneous gamma oscillations and/or gamma-band connectivity measured in resting state conditions are enhanced in patients with schizophrenia [105–113], especially in patients with positive or reality distortion symptoms (hallucinations and delusions) [114].

Revisiting the binding theory of mental representations [115], which assigns mental imagery to the interaction between distant brain regions (a synchronization in the gamma range) [116], such an increase or decrease in gamma oscillations may explain different phenotypes of schizophrenia. For example, the increase in gamma-band power and phase locking (i.e., connectivity) could be related to the emergence of new perceptual representations that are normally absent in healthy individuals, such as auditory hallucinations. In contrast, the decrease in gamma oscillations could be a sign of brain disintegration, and thus, the cause of the impairment or deterioration of normal cognitive functions in normal people. According to these observations, we consider schizophrenia a combination of different clinical presentations that are associated with dysconnectivity [117], not disconnectivity, of the brain, where the differential contributions of different dysconnectional (connection or disconnection) patterns of the patients’ brain determine their explicit clinical picture. This functional dysconnectivity is also confirmed by the aberrant underlying anatomical and cellular dysconnectivity [117–119].

In accordance with this assumption, cognitive impairments and negative symptoms in schizophrenia have been linked to reductions in gamma power and phase coherence, whereas positive symptoms are associated with increased gamma activity. In this regard, tACS may help with the diagnosis of and differentiation between different schizophrenic clinical syndromes, in addition to its role in treatment. Future research could exploit tACS-directed characterization of specific oscillatory endotypes in distinct schizophrenic presentations.

One of the main positive symptoms of Schizophrenia is the presence of auditory hallucinations that are sometimes treatment-resistant [120]. In EEG studies, auditory hallucinations in people with schizophrenia are significantly correlated with functional connectivity between both primary auditory cortices. Such enhanced functional connectivity is manifested in the phase synchronization between brain oscillations in both auditory cortices in the range of gamma-band frequencies [102, 121]. Interestingly, this phenomenon was further confirmed by structural changes using diffusion tensor imaging (DTI) [122]. These findings, in turn, support the interhemispheric miscommunication hypothesis of auditory hallucinations [123]. In the light of these discoveries, tACS was able to manipulate auditory perception in healthy participants by decoupling this interhemispheric connectivity. A remarkable finding was that the individual indigenous brain oscillations prior to stimulation dictated the resulting effects of tACS, favouring the use of individually tailored stimulation paradigms [124].

#### Clinical tACS studies

Seven publications studied the application of tACS in patients with schizophrenia. They ranged widely in terms of the evidence-based medicine hierarchy. Two case reports and a case series were reported, while the other four publications were clinical trials. Two of them were an open-label non-controlled trial and a single-blind randomized controlled trial. Finally, two publications comprised a well-structured double-blind randomized controlled study.

The two case reports were published by the same research group to examine the feasibility of tACS in schizophrenia. They were based on previous findings of reduced theta and gamma oscillation in relation to working memory, especially on the frontal region. One of them showed that one session of 20 min tACS applied to the left DLPFC (F3) and the left posterior parietal region (P3) in theta frequency (6 Hz), but not gamma (40 Hz), was able to improve performance in WM task [125]. The same tACS protocol was replicated in the other case report but for five consecutive days, resulting in improved working memory after 6 Hz-tACS, as well as an improvement in other cognitive domains. After 50 days of follow-up, these effects remained observable [126].

Similarly, the open case series in schizophrenia investigated the efficacy and safety of theta tACS, as theta waves are reduced in the frontal region. The study applied 20 daily tACS sessions for 4 weeks on working days in three subjects with clozapine-resistant schizophrenia. 4.5 Hz-tACS targeted both right and left DLPFCs with an amplitude of 2 mA for 20 min per session. Patients were assessed according to their psychiatric clinical symptoms (positive, negative and anxiety), and illness insight, as well as tACS adverse events. They showed a reduction in negative symptoms, anxiety and general psychopathology symptoms with improvement in illness insight [127].

Although the abovementioned case reports obtained results from only a few patients, they may support the feasibility of tACS in schizophrenia research. These effects might be long-term and frequency specific. Furthermore, the case series succeeded in reducing the schizophrenia symptoms and improving insight into treatment-resistant patients without serious side effects. However, well-structured controlled clinical trials are necessary to confirm these findings and to control for the placebo effect. Further research may need to verify modulation of the targeted oscillations using pre- and post-stimulation EEG recordings.

Motivated by the replicated finding of decreased frontal alpha activity in schizophrenia, the open-label non-controlled trial attempted to assess the safety and efficacy of alpha tACS on persistent delusions [128]. The trial recruited 12 patients with schizophrenia who exhibited persistent delusions, despite pharmacological treatment. All patients received two 20-min sessions per day separated by 3 h for 5 days. Nine of them received stimulations for five more days, and only one participant continued for four more days. tACS of 10 Hz was applied with 2 mA intensity via two electrodes over AFz and Cz. The study aimed to decrease persistent delusions by normalizing alpha oscillations in the medial prefrontal region. The patients were monitored by the Psychiatry Rating Scale (PSYRATS)—Delusions, the Scale for Assessment of Positive Symptoms (SAPS) and the Scale for Assessment of Negative Symptoms (SANS) and were followed up for one month. The results showed a reduction in the delusions, as well as positive and negative symptoms, which was maintained for one month. Interestingly, the patients tolerated the twice-daily regiment without serious side effects.

This study supports the feasibility of twice-daily stimulation and opens the door for the replication of frontal alpha tACS effects on persistent delusions as an add-on option. However, major limitations still exist that should be controlled for in further research. The sample size was small without a placebo control group, and patients were taking different medications, which might have affected the results.

One included publication was a single-blinded, randomized, controlled trial, which attempted to target frontal gamma oscillations that are reduced in schizophrenia. Ten patients with schizophrenia participated in three separate 20-min sessions at least three days apart. They randomly received one of three stimulation protocols: 40 Hz tACS, tDCS and sham stimulation. 40 Hz tACS was delivered on the left DLPFC with an amplitude of 2 mA. Meanwhile, they performed a working memory task before, during and after every stimulation session. The study did not report any significant effects of tACS on the task parameters or any serious side effects [129].

Given the small sample size, this study did not exclude the potential rule of gamma tACS on working memory in subjects with schizophrenia. More interestingly, the trial obtained neither pre- nor post-EEG recordings and did not utilize field modelling tools. The lack of an observed effect in response to tACS may be attributed to the difficulties of gamma stimulation per se or to different brain dynamics in patients, especially since the same group managed to improve working memory in healthy controls using the same stimulation protocol [130].

The remaining two publications on schizophrenia addressed the first well-structured randomized, double-blind, controlled clinical trial in patients with psychiatric disorders [4, 131]. It investigated the role of alpha tACS based on abnormalities in alpha oscillations over the frontal and temporal regions in schizophrenia. Twenty-two hallucinating participants with schizophrenia were randomized into three groups. While one control group received sham stimulation, the other active ones received either 10 Hz tACS (2 mA) or tDCS. Both were applied to the left frontal and temporal lobes. All groups received two 20 min sessions per day for five consecutive days. The primary outcomes were the improvement of auditory hallucinations calculated by the Auditory Hallucination Rating Scale (AHRS) and High-Density Electroencephalogram (hdEEG). Meanwhile, the secondary outcomes included the Positive and Negative Syndrome Scale (PANSS) and the Brief Assessment of Cognition in Schizophrenia (BACS). Only the group that received 10 Hz tACS exhibited modulation of functional connectivity, and enhancement of alpha oscillations and the 40 Hz auditory steady-state response (ASSR). Such enhancement correlated with a reduction in auditory hallucinations as measured by AHRS [131]. However, the primary and secondary clinical outcomes did not reveal significant effects.

This trial is not only the first clinical trial conducted in patients with schizophrenia, but it revealed the ability of tACS to modulate disturbed alpha oscillations in schizophrenia as well [72, 74]. Interestingly, such a modulation correlated with a reduction in auditory hallucinations as a clinical parameter. Despite this correlation, the clinical outcome did not reach significance. This might be attributed to the small sample size and/or the significant inter-group variation in age, especially since tACS showed the largest effect size for AHRS. Hence, this study requires further replications with a larger sample size and longer duration of follow-up to verify the efficacy of normalization of disturbed alpha oscillations in improving schizophrenia symptoms. Having reported no serious side effects, this study may justify the twice-daily stimulation protocol.

### Depression

Depression, similar to schizophrenia, shows a comparably complex picture of altered brain oscillations [132]. The electrophysiological features of depression manifest some heterogeneity, as well as a greater dependence on the frequency [132, 133]. Either in terms of the power or the coherence, the low-frequency bands (delta, beta and alpha) were enhanced during the resting-state in depression, in particular, alpha oscillations, which persist even after the transition from closed-eyes to open-eyes states [85, 133–136]. Additionally, the same waves indicated specific connectivity, interhemispheric asymmetries, and even probable prognostic patterns.

In the alpha-band synchrony, the dorsolateral prefrontal cortex (DLPFC) is more connected to the anterior cingulate gyrus [137] and temporal and parietal occipital regions [136]. Alpha-band interhemispheric comparisons revealed frontal alpha asymmetry (FAA) and parietotemporal alpha asymmetry, where the left hemisphere shows more alpha power and local synchrony than the right hemisphere [132, 138, 139]. Interestingly, enhanced alpha activity in depression was associated with better response to antidepressant therapies [140, 141]. Though theta waves were also mostly increased in depression, especially within the frontal short-range functional connections [142], in contrast to alpha waves, the enhanced frontal theta waves were correlated with decreased response [143].

Gamma oscillations differ significantly depending on the state, as they were reduced in the anterior cingulate cortex and in the frontal regions during the resting state and emotional tasks, respectively [144, 145]. Nevertheless, they were enhanced in the frontal and temporal lobes in response to spatial and arithmetic tasks [146]. Interestingly, gamma activity differentiated unipolar depression from bipolar depression according to its power in two different tasks: an auditory task augmented gamma ASSR power in unipolar depression, while an emotional task enhanced temporal and suppressed frontal gamma powers in unipolar depression with respect to bipolar depression [147–149]. Different antidepressant options exhibited either increased or decreased gamma oscillations: serotonergic medications, cognitive therapy and deep brain stimulations dampened them; and in contrast, noradrenergic drugs, ketamine and TMS induced them [145].

#### Clinical tACS studies

Three eligible articles examined the application of tACS in depression: one of them is a well-structured double-blind randomized controlled trial, while the other two present two case reports. In the double-blind randomized clinical trial, tACS targeted pathologically increased alpha waves on the left frontal region compared to the right frontal region (i.e., FAA). The study aimed to restore the frontal alpha oscillations by synchronously stimulating both frontal regions. Therefore, 32 patients with Major Depressive Disorder (MDD) were randomly recruited to three study groups: two groups were given two verum tACS protocols (10 Hz-tACS or 40 Hz-tACS) with an amplitude of 4 mA at Cz and 2 mA at F3 and F4, and the third group received active sham stimulation. The session took 40 min and was repeated for five consecutive days. The left DLPFC was the stimulation target area, aiming to improve clinical symptoms by retaining its normal alpha frequencies. The primary and secondary outcomes included the clinical symptoms applying Montgomery–Asberg Depression Rating Scale (MADRS) at 4-week follow-up and the normalization of alpha oscillations using hdEEG, respectively. Hamilton Depression Rating Scale (HDRS) and the Beck Depression Inventory (BDI) were chosen as exploratory outcomes. The study found no significant results regarding the primary outcome. In terms of MADRS and Hamilton Depression Rating Scale (HDRS), there were more responders at the two-week follow-up in the group that received 10 Hz-tACS. Concerning hdEEG, the same group showed significantly decreased alpha power in the left DLPFC on day 5. Moreover, there were no serious adverse events, manic shift or suicidal ideation induction [150].

This is the first well-structured clinical trial to record an effect of tACS stimulation in some patients with depression who were treated for two weeks by resetting the oscillatory brain disturbances. Such an effect was confined to alpha stimulation but not 40 Hz stimulation. This supports the idea that only stimulation at a specific frequency alters the oscillatory pattern and thus the behavioural outcome. However, this effect was not maintained after the 4-week follow-up and did not involve the primary outcome. Therefore, a larger number of stimulation sessions and longer follow-up periods should be encouraged since no serious adverse events were detected. Although no FAA was detected in the study sample at baseline, five sessions of 10 Hz-tACS managed to decrease the alpha power over the left frontal region. This might contradict that alpha tACS is supposed to enhance the alpha power as an immediate after-effect. Therefore, further research is needed to identify the possible mechanisms through which alpha tACS decreased the alpha power as a long-term effect. Given the small number of participants in this study, further research should be conducted with a larger sample size.

One of the included case reports on depression was an extension of the previous clinical trial where one participant received extra 12 weekly sessions of 10-Hz tACS. After the original study, the patient showed a response to the treatment without remission. After the 12 sessions, remission was achieved and maintained for at least a 2-month follow-up [151]. Despite being performed on a single participant, this study might support the feasibility of long-term tACS as a potentially safe tool in MDD treatment research.

The other case report examined the effect of frontal tACS at the gamma frequency band, which is reduced frontally in depressed patients [152]. A pregnant female MDD patient was recruited into the study at week 6 of pregnancy. She received 40 Hz-tACS of 2 mA over both DLPFCs (F3 and F4) for 20 min per session. After nine weekly stimulation sessions, she was followed-up at two weeks and three months (week 27 of the pregnancy) after the intervention. Her depression condition was monitored by HDRS, BDI, Positive and Negative Affect Schedule (PANAS) and Trail Making Test (TMT). The study only reported phosphenes without serious side effects. At the end of the nine stimulation sessions and 2-week follow-up, the patient showed improvement in her symptoms and experienced remission after three months. However, it is noteworthy that the same patient showed remission on tDCS used to treat a previous depressive episode. Therefore, the outcome could be attributed to a placebo effect or the stimulation per se, especially because no EEG recordings were done throughout the study [152]. Nonetheless, this case report may encourage further application of tACS in research on pregnant patients as a relatively safer option compared to pharmacotherapeutic agents.

### Obsessive–compulsive disorder

An enormous body of evidence has shown decreased alpha activity in resting state, task-based and symptom-provocation studies in obsessive–compulsive disorder (OCD), especially in frontal areas (hyper-frontality), reasoning the mental overactivity in OCD [139, 153–158]. Interestingly, the location of the resting alpha reduction was subtype-specific: within the frontotemporal areas in doubting OCD subtype; while over the parietooccipital regions in the checking subtype [159]. Unlike depression, alpha asymmetry is not a stable finding in OCD. Nevertheless, it is more pronounced in the doubting OCD with decreased left alpha waves [139, 159]. The decreased alpha activity during the task state may reflect the augmented readiness and/or the preoccupation of OCD patients by obsessions [160]. Surprisingly, alpha synchronization in working memory was reinforced, proposing a compensatory mechanism to inhibit irrelevant information [139, 161].

Theta and delta power, especially in frontal regions [157, 162–165], are augmented in OCD [154, 157, 159, 166, 167], where theta augmentation, similar to in depression, is correlated with poor response to treatment [168, 169]. The findings of beta oscillations were broadly inconsistent [169], but it is thought to be frontally elevated, originating from the anterior cingulate gyrus [166, 167]. Moreover, the frontal beta showed interhemispheric asymmetry with increased activity on the left side [166]. Although gamma-band oscillations are not well studied in OCD, they are thought to be generally decreased [85]. TACS might be used to normalize the altered oscillations, to potentiate the compensatory changes, and to differentiate subtypes in OCD.

#### Clinical tACS studies

Only one publication on OCD was included, which discussed a case series of seven treatment-resistant OCD patients. The authors suggested that “DLPFC activity in OCD might be pathologically reduced”, and thus gamma tACS might lead to its activation. 40 Hz tACS was administered at both frontotemporal sites (Fp1-T3 and Fp2-T4) to stimulate DLPFC. Patients received three 20-min sessions per week, while the duration of treatment varied across patients from two to seven weeks. All patients were clinically evaluated via the Yale-Brown Obsessive–compulsive Scale (YBOCS) and Clinical Global Impression Scale (CGI) on day 1 before tACS stimulation, and 28 and 56 days later. Three patients were followed up for 1 year. Symptoms improved in all patients, and the improvement lasted throughout the follow-up duration [3].

The study successfully reduced clinical symptoms of OCD, opening the door for further tACS research in OCD. However, it lacks EEG recordings to evaluate the effect of gamma stimulation on DLPFC. More importantly, it addressed only seven patients with different ages and medication parameters, and the placebo effect cannot be ruled out. Further double-blind controlled studies are necessary to investigate the reproducibility of such an empirical finding.

### Bipolar disorder

Generally, alpha oscillations are inhibited in bipolar patients in many aspects: both resting and evoked alpha waves, with eyes closed or open, and in euthymic or manic participants [170–172]. In contrast, theta and delta oscillatory activity is enhanced [170, 173–175]. Likewise, an enhancement in the beta power response to different stimuli was recorded, which differentiated bipolar disorder from schizophrenia [171, 176–178]. Nonetheless, the beta synchronization was decreased at rest and in response to an auditory stimulation [179, 180]. Both manic and euthymic patients showed a decreased gamma coherence as evoked by diverse stimuli [96, 148, 181–183]. In summary, the oscillatory changes in bipolar disorder curb the resting-related alpha waves, as well as beta and gamma synchronization, leading to restrained connectivity between brain regions. These two principal findings may explain the disturbed racing thoughts and the distractibility in patients [184] and could be targeted by tACS for normalization. So far, no clinical study investigating the role of tACS in bipolar disorder has been reported according to the systematic search.

### Attention-deficit/hyperactivity disorder (ADHD)

Only one included article addressed ADHD as a single-blind randomized controlled trial. In ADHD, the target P300 amplitude shows some reduction, which is associated with typical cognitive performance deficits in ADHD. Therefore, the trial investigated the role of tACS in potentiating the target P300 amplitude as disturbed brain oscillations in the theta and delta range. In addition, it aimed to improve cognitive performance in patients with ADHD by such P300 amplification. Therefore, EEG was used to examine brain activity in 18 patients with ADHD underlying a visual oddball paradigm. During the task, 1 mA tACS was applied for 20 min through multiple electrodes on the central, parietal and temporal lobes. The stimulation timing and frequency were personalized so that the stimulation peaks could coincide with P300 oscillation peaks to amplify them. The study found P300 amplitude to be significantly augmented in the verum group compared to the sham condition. Interestingly, this P300 amplification was accompanied by a behavioural improvement in task performance. No serious adverse events were reported [185].

This clinical trial is not only the first study to support research on the role of tACS in ADHD, but it also substantiates tACS applicability to modulate event-related potentials for clinical relevance. Nonetheless, future studies could replicate it with more patients and compare tACS effects in patients and healthy controls, especially because a similar stimulation protocol did manage to alter P300 parameters in healthy participants [186]. However, this study paved the way for the feasibility of tACS to modulate event-related potential components with subsequent behavioural improvement without serious side effects.

### Dementia

To the best of our knowledge, there is no available study using tACS as a treatment in either Alzheimer’s disease (AD) or dementia. Only a single included study examined the role of tACS as a prognostic factor in predicting the progression of patients with mild cognitive impairment (MCI) to AD. The study was based on findings of decreased gamma-band connectivity in AD [187–192] and increased local gamma-band power in contrast to MCI during resting and task conditions [193]. The study questioned whether the response to gamma tACS could differentiate MCI from AD and predict the progression of MCI to AD. The authors recruited 35 AD and 25 MCI individuals, as well as 27 age-matched healthy participants, and followed them up for 2 years. On a weekly basis, each participant randomly received both sham stimulation over the left primary motor area (M1) and five verum stimulation sessions. Gamma-band tACS was applied for 10 min to one of the following sites per verum session: M1, premotor area (PMA), supplementary motor area (SMA), DLPFC, or the dorsomedial prefrontal cortex (DMPFC). Stimulation of 1 mA was set to vary continuously and randomly in a range between 40 and 120 Hz at steps of 20 Hz, with zero-degree phase-lag. EEG was recorded before and after each session in addition to a follow-up EEG after 2 years.

tACS caused enhancement of gamma-band oscillations and a clinical improvement according to different neuropsychological tests in MCI patients. AD patients, however, showed neither. After 2 years, a group of MCI patients progressed to AD. Interestingly, this group did not report any tACS after-effects before the 2-year follow-up period [194]. The sessions were well tolerated by the patients.

This is the first publication to report the use of tACS on dementia. Its results might help to identify the potential role of tACS in the differential diagnosis of MCI and AD and in prognosis prediction for MCI. Nevertheless, it is still far from providing a biomarker for dementia progression given the discrepancy between connectivity within and local power of gamma oscillations. Further research should replicate this study in larger sample sizes to verify the reproducibility of these findings. Moreover, follow-up time points should be encouraged with EEG recordings to obtain electrophysiological markers of the disease progression.

## General discussion

To the best of our knowledge, this is the first systematic literature search to report studies that apply tACS in clinical psychiatric research. tACS publications on five different psychiatric disorders were reported. First, in schizophrenia, frontal alpha tACS was promising as an add-on treatment for persistent delusions [128] and was capable of normalizing disturbed alpha oscillations correlated with a decrease in auditory hallucinations [4, 131]. On the other hand, frontal gamma tACS has not shown effects so far [125, 129]. Case reports revealed that frontal theta tACS could be helpful for improving clinical symptoms, even in schizophrenia with clozapine resistance [125–127]. Second, in depression, bifrontal alpha tACS was able to normalize alpha oscillations and concurrently showed a higher response rate for at least two weeks [150]. Two case reports showed that tACS might be safely tolerated, even for a large number of sessions or during pregnancy [151, 152]. Third, a case series in OCD with seven patients revealed an empirical improvement in symptoms after frontal gamma tACS [3]. Fourth, a single-blind trial managed to amplify P300 in ADHD patients with subsequent improvement in working memory task [185]. Finally, frontal gamma tACS might support a potential role for tACS as a diagnostic or prognostic tool in MCI and AD [194]. All the studies reported neither serious side effects nor exacerbation of clinical symptoms (i.e., manic shift or worsening of the clinical phenotype) [3, 4, 125–129, 131, 150–152, 185].

Despite the very limited number of studies applying tACS in psychiatry, its efficiency seems promising in the psychiatric field keeping in mind the following considerations: (1) some studies have recruited treatment-resistant patients, and managed to diminish their symptoms [3, 127]; (2) the relatively good safety profile of tACS may advocate twice-daily stimulations, use in pregnancy, or large stimulation doses without fear of clinical worsening [128, 131, 150–152]; (3) the novelty of its treatment approach targeting specific alterations of brain functions at specific locations makes it significantly differ from conventional treatment strategies, such as pharmacotherapy. Although psychotropic drugs are widely accepted and approved in psychiatry, they still pose substantial problems with resistance, compliance and safety profile [195–197]; (4) tACS could help to target baseline oscillations, as well as event-related potentials [185]; (5) utilization of tACS should not be restricted to therapeutic purposes. The reactivity of the brain circuits to the stimulation protocol could serve as a possible tool to determine the diagnosis, classification or prognosis of psychiatric disorders, for instance, in patients with MCI [194]. However, tACS in psychiatric research is still far from being approved as a reliable tool for the management of psychiatric conditions. Further research is necessary to replicate these findings and to answer important questions.

An intriguing problem might be whether disturbed brain oscillations are causally related to the disorder or represent just an association. Moreover, it is not yet clear whether tACS can be investigated as a stand-alone therapeutic tool or rather an add-on option to target specific residual symptoms. The concomitant use of drugs while applying tACS could complicate future research. Drug-naïve and drug-non-compliant patients could be recruited to rule out effects of the drugs or to prove a synergistic or antagonistic effect between both tACS and pharmacotherapy. Along with this, the therapeutic role of tACS should be precisely defined with respect to whether it can improve the whole clinical picture of psychiatric illness [150] or is confined to targeting a specific modality of symptoms [185] that did not respond to other therapeutic options. Referring to the phenomenon of Arnold tongues, the weak tACS current could entrain the brain activity associated with a certain brain state when the stimulation targets these brain oscillations at their frequency. Hence, a certain brain state may be induced in participants to maximize these oscillations, so they can easily be entrained by tACS (i.e., state-dependent stimulation) [198]. In that sense, tACS could be initially used to target a pattern of brain oscillations driven by a single modality or symptom of psychiatric disorders. This was shown to be possible in targeting P300 amplitude driven by WM in ADHD and gamma oscillations induced by WM in MCI [185, 194].

The discrepancy of brain dynamics and response to tACS between healthy participants and patients constitutes an enigma that should be considered during replication of similar stimulation protocols. Healthy participants may be more susceptible to specific tACS stimulation protocols than patients with psychiatric disorders [129, 130] or vice versa [185, 186]. Similarly, acute and chronic patients may show distinct tACS responses, especially tACS after-effects, which necessitates long-term plasticity that might be affected in chronic patients [199]. Future research may try to determine potential responders to tACS based on their electrophysiological markers or clinical phenotype.

In light of these future research questions, several points could be highlighted. The more individualized the stimulation protocol is designed, the more effectively it normalizes the disturbed oscillations, for example, in the schizophrenic case reports and the ADHD study [125, 126, 185]. tACS effects do not depend on the direct application of a specific frequency per se but on modulation of the pre-existing endogenous oscillations that are coupled with the specific brain state. Accordingly, it might be helpful to personalize future stimulation paradigms, by individually defining disturbed EEG patterns regarding frequency and localization of interest prior to stimulation and then tailoring the stimulation parameters (frequency and location of stimulation) accordingly [200, 201]. Such personalisation of the stimulation protocols stands in accordance with previous findings of the heritable electrophysiological endophenotypes associated with some psychiatric disorders [202, 203]. For this, tACS modelling techniques might be recommended to optimize stimulation parameters and obtain better results [13]. Overall, in the same context, electrophysiological recording via EEG or MEG could be valuable to observe achievement of the desired change in brain electrical activity and to individualize the stimulation protocol [204]. Finally, multicentre double-blind clinic trials could be fostered to investigate larger sample sizes. This may also facilitate the utility of different stimulation protocols in different patients or within the same individuals to rule out a stimulation effect per se [4, 125, 131, 150] and to question the potential synergism of different stimulation parameters, respectively (for more technical guidance: [12, 205–207]).

Despite the relative safety profile of tACS, several side effects could coincide with the stimulation period: phosphenes, dizziness, headache and skin sensations, such as tingling, itching, etc.[205]. Phosphenes tend to be more frequent upon frontal montages due to retinal stimulation. On the other hand, dizziness is more common in posterior montages due to vestibular stimulation. Skin adverse events, as well as phosphenes, frequently co-occur with higher frequencies and/or intensities [208–210]. Headache could outlast the stimulation phase, especially with longer stimulation duration [211, 212]. In contrast to tDCS, tACS induces less serious (e.g., epilepsy) and less persistent (e.g., burn and dermatitis) adverse events [208, 213].

To display a balanced overview of the current insufficient knowledge on tACS, two major limitations should be presented: (1) some studies failed to show an effect in both healthy participants and patients, and (2) entrainment of brain oscillations is confounded by other proposed mechanisms of action. The individual functional as well as the structural variability of the brain, the wide range of stimulation parameters and technical difficulties could explain why tACS failed to alter the behavioural outcomes [67, 129, 186, 214, 215]. In this regard, strict modelling techniques and peri-stimulation EEG recordings could decipher the inability of tACS to induce electrophysiological or behavioural outcomes. Consistently, this limitation is complicated by the obstacle of stimulation artefact rejection in EEG or MEG [216, 217]. Future research may be needed to identify optimal tACS parameters to ensure a consecutive effect.

A second major drawback of tACS is the indirect mechanisms of action rather than direct entrainment of endogenous brain oscillations. Stimulation of peripheral nerves and the retina could account for the entrainment of brain oscillations [213, 218–222]. Similarly, cranial electrotherapy stimulation, a close alternating current stimulation tool applied on the forehead and mastoids, induces electrical brain changes via direct stimulation of cranial nerves [223, 224]. Although this hypothesis of the retina and peripheral somatosensory stimulation cannot be fully excluded, evidence supports the direct causality of tACS to entrain brain oscillations [20, 222, 225]. Consistent with this conclusion, tACS revealed frequency-, phase- and montage- and state-specific effects [67, 198, 222, 226]. Further research could try to estimate the contribution of these indirect mechanisms to the whole tACS using modelling techniques, as well as active control groups [222].

## Conclusions

tACS, a unique form of NIBS, results in both online and offline brain changes by entraining brain oscillations and inducing neuroplasticity, respectively. It has been extensively used to alter electrophysiological brain activity, and thus cognitive functions in healthy participants. Similarly, disturbed brain oscillations in psychiatric conditions may constitute a potential target for modulation by tACS without major adverse events. Its first few applications in psychiatry seem promising and encouraging for more research to discover its full potential with respect to therapeutic and diagnostic roles. Given its safety profile, these first studies may support tACS feasibility in altering disturbed brain oscillations, thus improving behavioural outcomes. However, further well-structured double-blind controlled trials with larger sample sizes and longer follow-up durations are still needed to replicate the current findings. They may help to detect response predictors and control for various confounding factors. In this regard, electrophysiological recordings, as well as modelling techniques, are encouraged to optimize stimulation protocols and to detect possible factors contributing to the effects of tACS.

## Abbreviations

AD: Alzheimer’s disease
ADAS-Cog: The 11-item cognitive subscale of the Alzheimer’s Disease Assessment Scale
ADHD: Attention deficit hyperactivity disorder,
AHRS: Auditory Hallucination Rating Scale
ASSR: Auditory steady-state response
BACS: Brief assessment of cognition in schizophrenia
BDI: Beck Depression Inventory
CES: Cranial electrotherapy stimulation
CGI: Clinical Global Impression Scale
DLPFC: Dorsolateral prefrontal cortex
DMPFC: Dorsomedial prefrontal cortex
DTI: Diffusion tensor imaging
EEG: Electroencephalography
FAA: Frontal alpha asymmetry
HAMA: Hamilton Anxiety Rating Scale
hdEEG: High-density electroencephalography
HDRS: Hamilton Depression Rating Scale
LTD: Long-term-depression
LTP: Long-term-potentiation
M1: Primary motor
MADRS: Montgomery–Asberg Depression Rating Scale
MCI: Mild cognitive impairment
MDD: Major depressive disorder
MEG: Magnetoencephalography
NIBS: Non-invasive brain stimulation
OCD: Obsessive–compulsive disorder
PANAS: Positive and Negative Affect Schedule
PANSS: Positive and Negative Syndrome Scale
PMA: Premotor area
PSYRATS: Psychiatry rating scale
SANS: Scale for the Assessment of Negative Symptoms
SAPS: Scale for the Assessment of Positive Symptoms
SMA: Supplementary motor area
STDP: Spike-timing-dependent plasticity
SUMD: Scale to Assess Unawareness of Mental Disorder
tACS: Transcranial alternating current stimulation
tDCS: Transcranial direct current stimulation
TMS: Transcranial magnetic stimulation
TMT: Trail Making Test
WM: Working memory
YBOCS: Yale–Brown Obsessive–compulsive Scale
